# Supplementary material for: “Thought I’d Share First” and Other Conspiracy Theory Tweets from the COVID-19 Infodemic: Exploratory Study
Source: JMIR Public Health Surveill. 2021 Apr 14;7(4):e26527. doi: 10.2196/26527 (PMC8048710; doi:10.2196/26527)

## Multimedia Appendix

**Figure S1. Sentiment comparison for “5G” data by label.** Color indicates the number of tweets found on that date with corresponding net sentiment score.
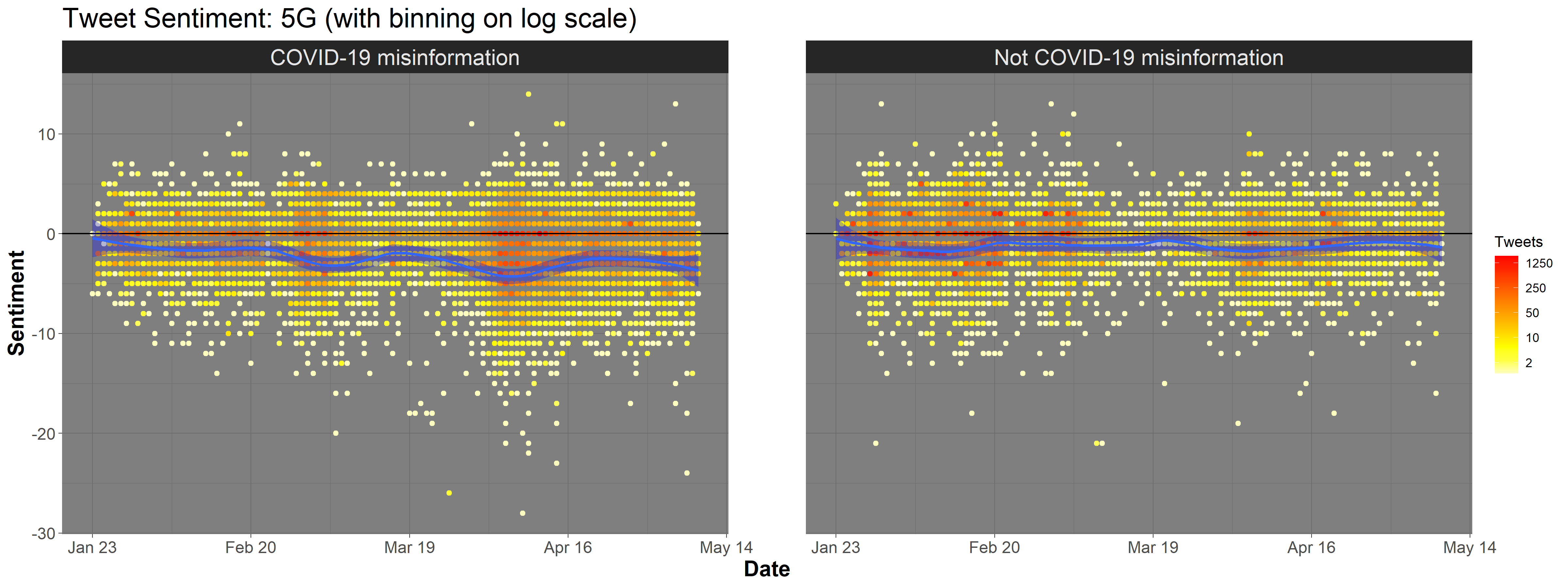


**Figure S2. Sentiment comparison for “Lab” data by label.** Color indicates the number of tweets found on that date with corresponding net sentiment score.
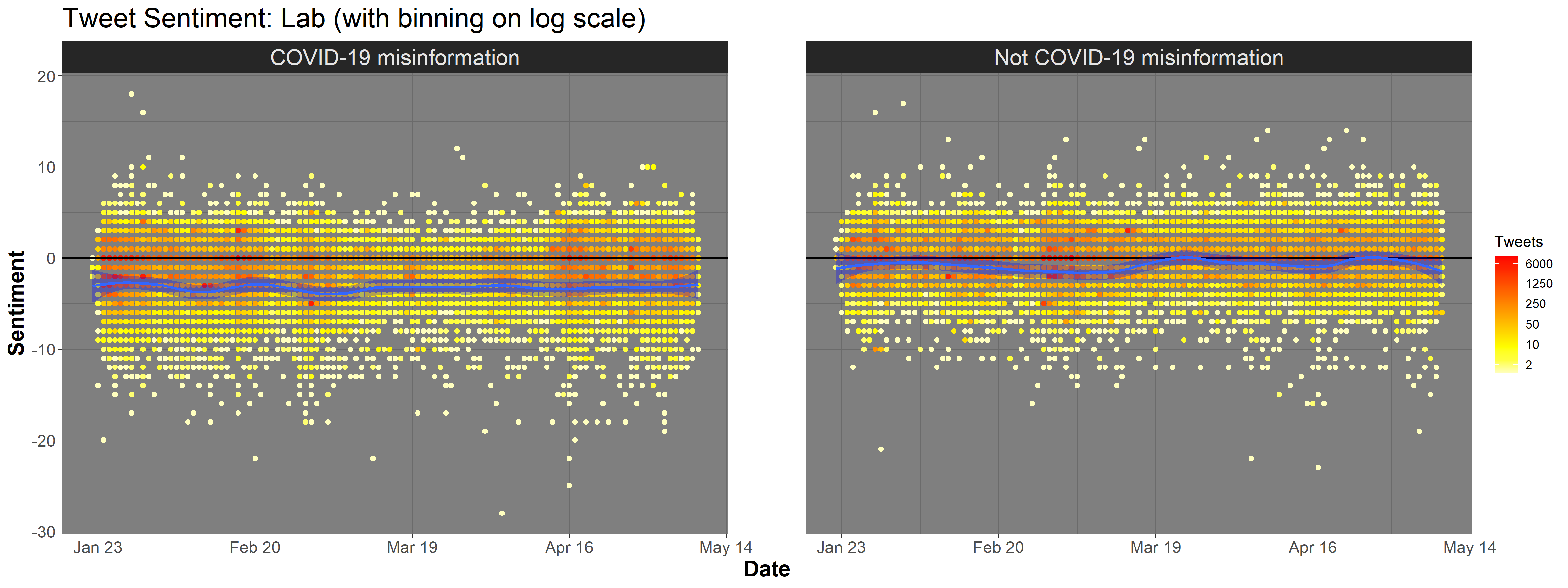


**Figure S3. Sentiment comparison for “Vax” data by label.** Color indicates the number of tweets found on that date with corresponding net sentiment score.


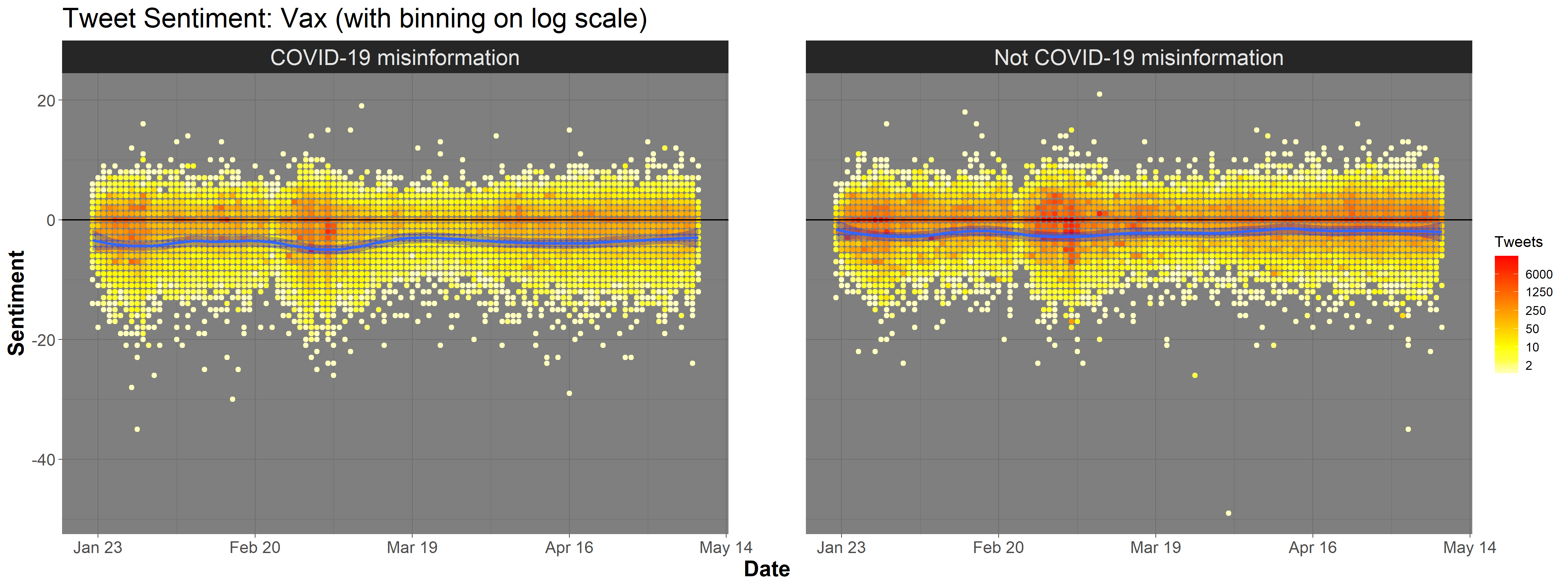


**Figure S4: Word cloud and topic evolution for topics of tweets related to 5G technology. Top Panel**: Word evolution (change in word importance) over time for tweets. Here, the x-axis represents time, the y-axis shows important words, and the color represents importance of words with darker color denoting higher importance. **Bottom Panel**: Word clouds for each topic. Word size corresponds to word weight (larger words have a higher weight).


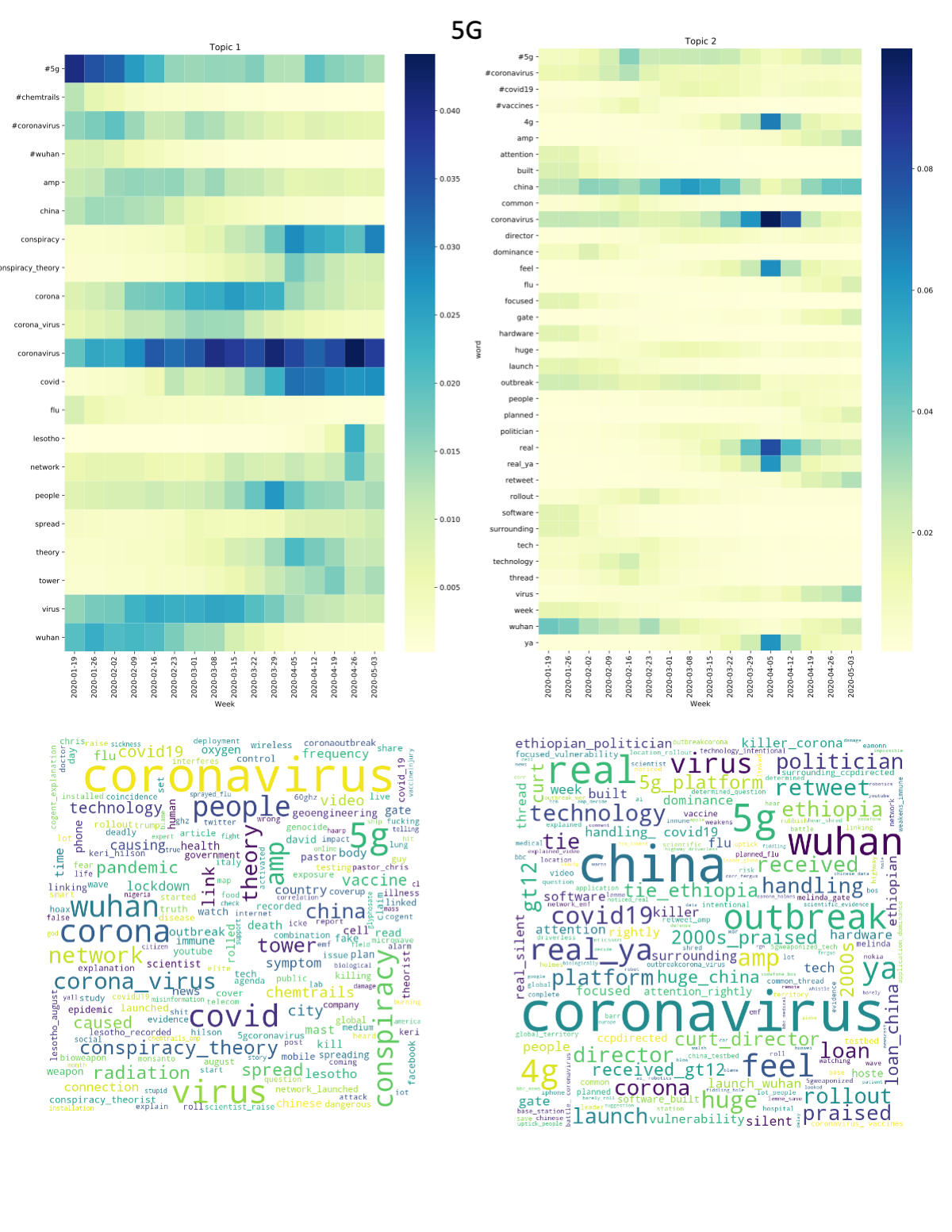


**Figure S5: Word cloud and topic evolution for topics of tweets related to laboratory origins of the virus.** **Top Panel**: Word evolution (change in word importance) over time for tweets. Here, the x-axis represents time, the y-axis shows important words, and the color represents importance of words with darker color denoting higher importance. **Bottom Panel**: Word clouds for each topic. Word size corresponds to word weight (larger words have a higher weight). Words such as “biosafety”, “biowarfare”, “warned”, and “laboratory” early in the pandemic suggested that people were discussing a malicious laboratory release [63], whereas later words like “escaped”, “evidence”, and “originated” corresponded to theories that focused on an accidental release of the virus from a laboratory.


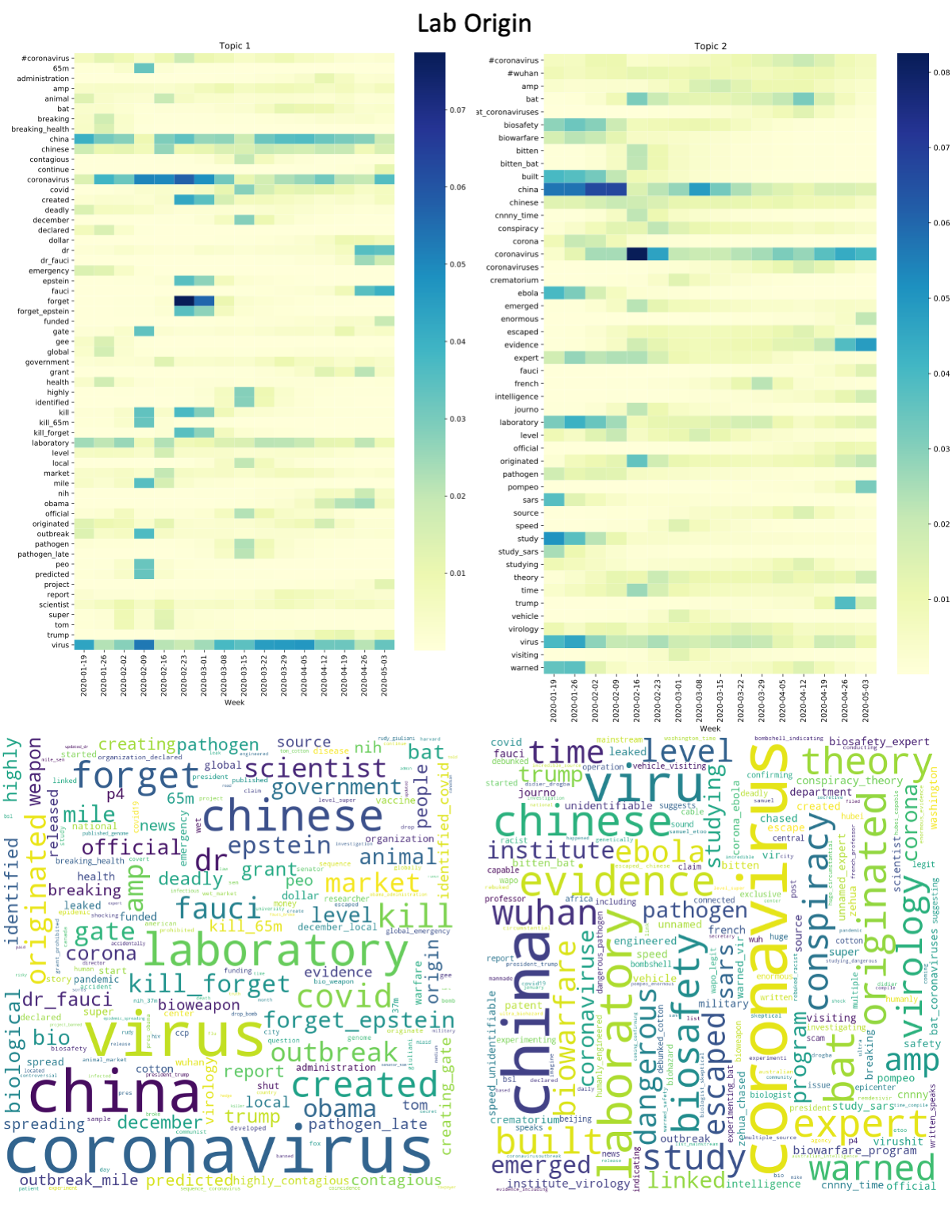


**Figure S6. Word cloud and topic evolution for topics of tweets related to vaccine discussion.** **Top Panel**: Word evolution (change in word importance) over time for tweets. Here, the x-axis represents time, the y-axis shows important words, and the color represents importance of words with darker color denoting higher importance. **Bottom Panel**: Word clouds for each topic. Word size corresponds to word weight (larger words have a higher weight). The term “bakker” in Topic 1 refers to the tele-evangelist Jim Bakker who promoted myths about possible COVID-19 cures, including promoting the use of colloidal silver on his show. He was given a cease-and-desist letter from the federal government as a result [54].


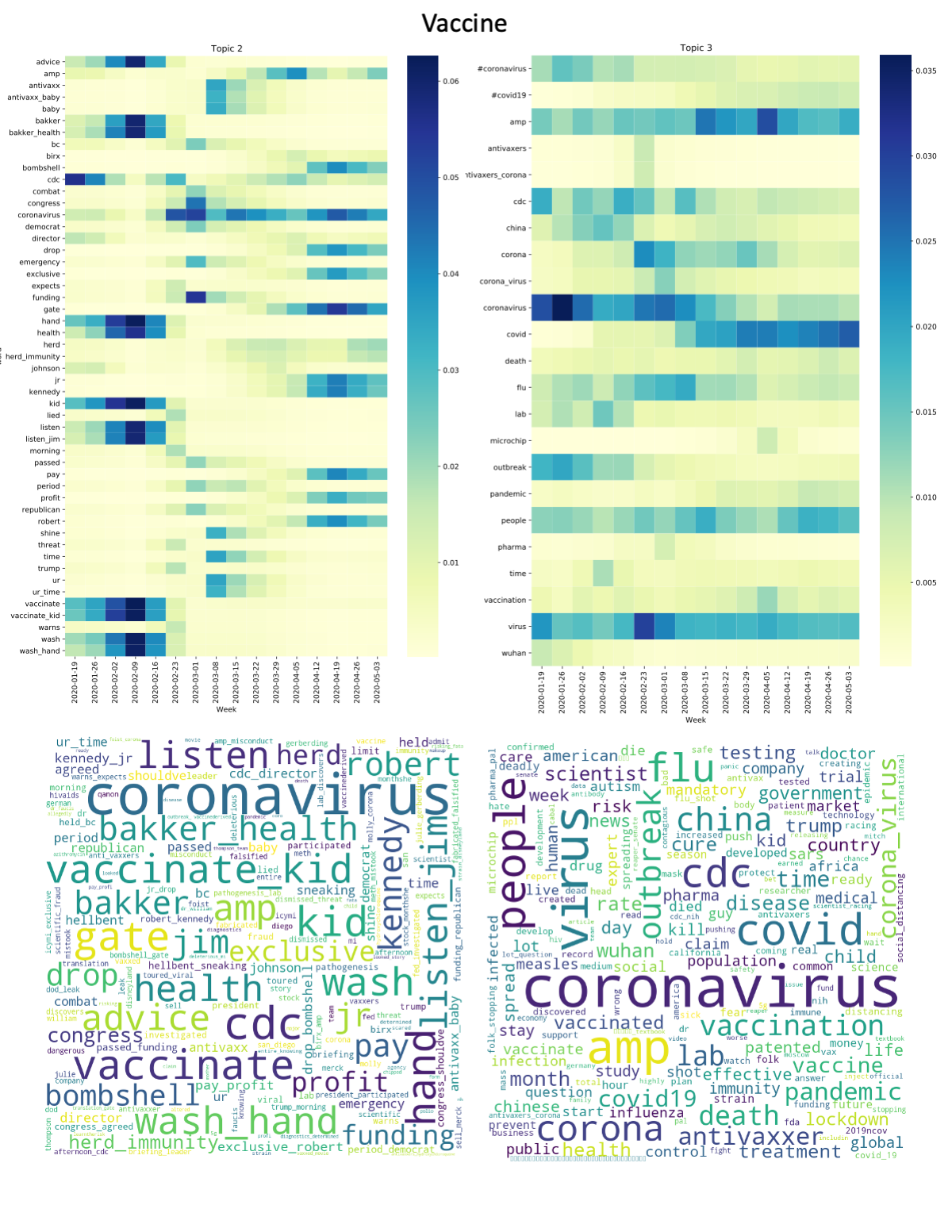

Supplement: Multimedia Appendix 1 [file publichealth_v7i4e26527_app1.docx]
